# Improved Virtual Gyroscope Technology Based on the ARMA Model

**DOI:** 10.3390/mi9070348

**Published:** 2018-07-11

**Authors:** Jinlong Song, Zhiyong Shi, Lvhua Wang, Hailiang Wang

**Affiliations:** Department of Vehicle and Electrical Engineering, Army Engineering University, Shijiazhuang 050003, China; sjzsong_jl@163.com (J.S.); 18222885931@163.com (L.W.); m18731697828@163.com (H.W.)

**Keywords:** MEMS, virtual gyroscope, random error, autoregressive moving average (ARMA) model

## Abstract

In view of the large output noise and low precision of the Micro-electro-mechanical Systems (MEMS) gyroscope, the virtual gyroscope technology was used to fuse the data of the MEMS gyroscope to improve its output precision. Random error model in the conventional virtual gyroscopes contained an angular rate random walk and angle random walk ignoring other noise items and the virtual gyroscope technology can not compensate all random errors of MEMS gyroscope. So, the improved virtual gyroscope technology based on the autoregressive moving average (ARMA) model was proposed. First, the conventional virtual gyroscope technology was used to model the random error of three MEMS gyroscopes, and the data fusion was carried out by a Kalman filter to get the output of the virtual gyroscope. After that, the ARMA model was used to model the output of the virtual gyroscope, the random error model was improved with the ARMA model, and the Kalman filter was designed based on the improved random error model for data fusion of the MEMS gyroscopes. The experimental results showed that the 1σ standard deviation of the output of the virtual gyroscope based on the ARMA model was 1.4 times lower than that of the conventional virtual gyroscope output.

## 1. Introduction

With the advantages of small volume and low cost, MEMS gyroscopes are widely used in aviation, aerospace, and other navigation fields [1,2]. But, the precision of the MEMS gyroscope is lower than the traditional gyroscope because of the limit by the current level of technology. So, it is particularly important to improve the detection accuracy of the MEMS gyroscope by the use of virtual gyroscope technology [3].

The Jet Propulsion Laboratory (JPL) of the US National Space Agency (NASA) simulated 4 gyroscopes for 111 h, the performance of the gyroscope is increased by 173 times with the Kalman filter fusion, and the virtual gyroscope technology has been proposed [4]. The virtual gyroscope technology is used to carry out static and dynamic compensation of MEMS gyro random error [5]. The dynamic modeling of the angle, angular velocity, and angular acceleration is carried out, which increases the compensation precision of the virtual gyroscope in dynamic state [6]. The angular acceleration is modeled as a first order time correlation process, and the dynamic modeling of angular velocity is carried out to further improve the compensation precision of the dynamic virtual gyroscope [7]. The one order autoregressive model (AR (1)) is used to model the angular velocity and the optimal estimation process is improved, which reduces the complexity of data processing and improves the dynamic compensation performance of the virtual gyroscope technology [8]. The Allan variance and adaptive neuro-fuzzy inference system (ANFIS) are combined to carry out the weight of the MEMS gyroscope array. The output of each gyroscope is multiplied by weights, and the virtual gyroscope output is obtained by summing the product [9]. The Kalman filter is used to realize data fusion of 6 MEMS gyroscopes, which improves the output precision of MEMS gyroscope [10]. The data fusion experiments of six gyroscopes with relatively less correlation are carried out, and the influence of noise parameter on the fusion results is analyzed [11]. There are four MEMS gyroscopes integrated on one chip, which improves the correlation between the gyroscopes [12]. The relationship between correlation coefficient and data fusion results of MEMS gyroscope array is fully analyzed [13]. The design of the MEMS gyro array with 3 × 3 plane structure is carried out [14]. The data fusion test is carried out by using 72 MEMS gyroscopes, and the relationship between the number of gyroscopes and the effect of the fusion is analyzed [15].

Data fusion can be achieved by using the correlation between the gyroscope random errors in the virtual gyroscope technology, and the MEMS gyroscope random error can be compensated to a certain extent. The current virtual gyroscope technology has the following characteristics: when static, the compensation precision of the virtual gyroscope technology is high, but the compensation ability of the virtual gyroscope technology is very limited when dynamic. In addition, the current virtual gyroscope technology uses different Kalman filters in static and dynamic state, so it is necessary to use different methods to fuse static and dynamic data respectively. Because it is difficult to model the true value of the angular rate in a dynamic state, the effect of the true value of angular rate is usually offset by the output difference of gyroscope, which causes the error of the Kalman filter equation to describe the actual problem, which leads to the lower compensation precision. When static, the true value of the angular rate is 0, the true value is modeled as noise, and the modeling accuracy is high, so the accuracy of error compensation is good. But, the random error modeling in virtual gyroscope technology only takes the angle random walk and the angular rate random walk, neglects other noise terms, so the compensation precision of the virtual gyroscope technology still has some space to improve.

In this paper, the ARMA model and virtual gyroscope technology are combined, and an improved virtual gyroscope technology based on ARMA model is proposed. This method can further improve the random error compensation ability of virtual gyroscope technology in static state. The conventional virtual gyroscope technology is used to model the random error of MEMS gyroscope, and the data of three MEMS gyroscopes are fused. Then the ARMA model is used to model the virtual gyroscope output. The improved random error model is established by combining ARMA model and random error model in conventional virtual gyroscope technology. Based on the modified random error model, the Kalman filter is designed for data fusion to further compensate the residual drift in virtual gyroscope output. Finally, the feasibility of the proposed method is verified by experiments.

## 2. Allan Variance Analysis

**Figure 1:**
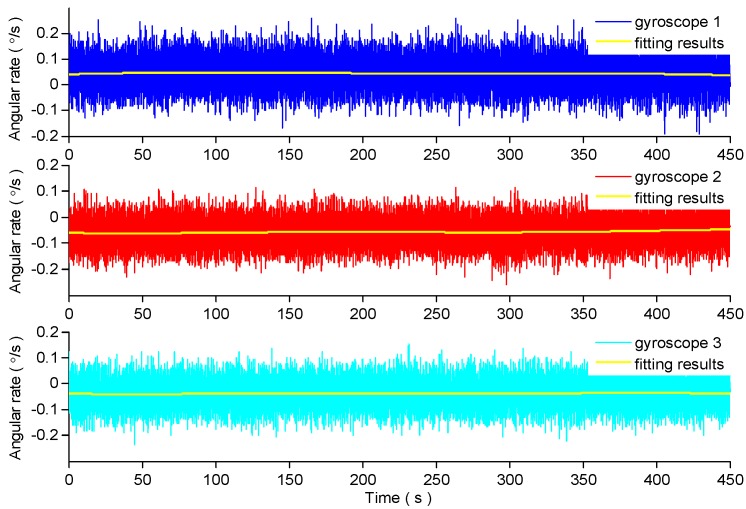
The original output signal of the three gyroscopes.

**Figure 2:**
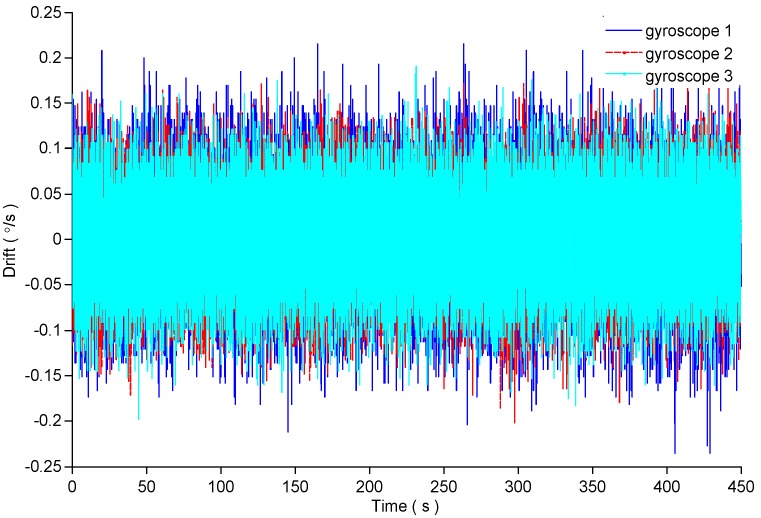
The random drift of the three gyroscopes.

Because the MEMS gyroscope is sensitive to temperature, the MEMS gyroscope array is installed on the test-bed with zero input of the external turntable for 1 h to achieve the working temperature of the MEMS gyroscope. The bandwidth of the used gyroscope is 40 Hz, so the sampling frequency is 100 Hz according to the sampling theorem. The total sampling time is 450 s. The static output of the three gyroscopes is shown in Figure 1. The 3-order polynomial fitting method is used to eliminate the trend item in the output of each gyroscope. The fitting results are shown in Figure 1, and the random drift of the three gyroscopes is shown in Figure 2.

Allan variance is a method to analyze the stability of signal frequency in the time domain. It was first proposed by the David Allan of the United States Standard Bureau, which is mainly used to determine the power spectrum of the frequency fluctuation of the atomic clock [16]. The statistical characteristics of the random drift of the MEMS gyroscope are similar to the statistical characteristics of the atomic clock, so many scholars also identify the random errors of the MEMS gyroscope using the Allan variance method. It is assumed that the MEMS gyroscope is sampled with the sampling interval of $\tau_{0}$, and *N* sampling points are obtained. Dividing the sampling sequence {ω} into *K* groups, *K = N*/*M,* and each group contains *M* data, the total sampling time for each group is related time τ, $\tau =M\tau_{0}$. The mean of each group is shown in Equation (1).

(1) $${\bar{\omega }}_{k}(M)=\frac{1}{M}\sum_{i=1}^{M}\omega_{(k-1)M+i},(k=1,2,\cdots K)$$

The Allan variance is defined as shown in Equation (2).

(2) $$\sigma_{A}^{2}(\tau )\cdot \frac{1}{2}\langle {\left({\bar{\omega }}_{k+1}(M)-{\bar{\omega }}_{k}(M)\right)}^{2}\rangle =\frac{1}{2(K-1)}\sum_{k=1}^{K-1}{\left({\bar{\omega }}_{k+1}(M)-{\bar{\omega }}_{k}(M)\right)}^{2}$$

**Figure 3:**
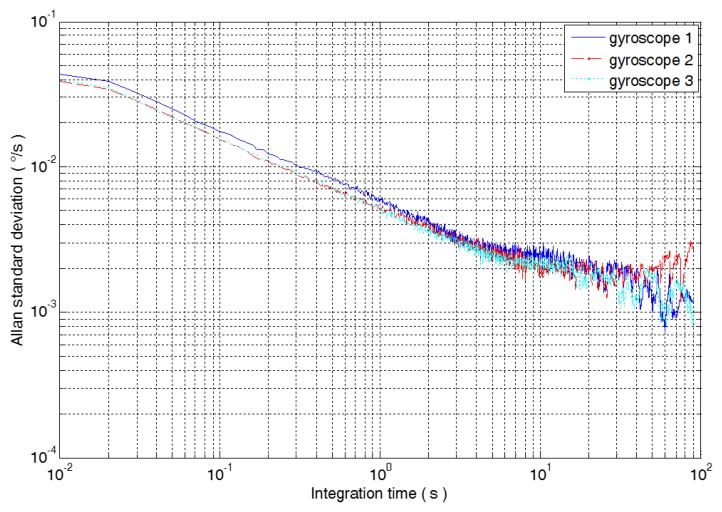
The Allan standard deviation curve of the three gyroscopes.

In the equation, <> represents the overall mean of the data. The Allan standard deviation curves of the 3 MEMS gyroscopes are shown in Figure 3.

The MEMS gyroscope random errors mainly include such as angular random walk (ARW), bias instability (BI), rate random walk (RRW), rate slope (RR), and quantization noise (QN). Other noises such as Markov noise (MN), which is also known as exponential correlation noise and sinusoidal noise (SN), are very small in the MEMS gyroscope errors, so it is not included in the error index in this paper. Due to the difference of gyroscope type and testing environment, there may be random noises of various components in the output data. It is considered that each noise is independent of each other and the Allan variance can be expressed as the sum of all the random errors. The Allan variance is expressed as Equation (3) [5].

(3) $$\sigma^{2}(\tau )=\sigma_{ARW}^{2}(\tau )+\sigma_{BI}^{2}(\tau )+\sigma_{RRW}^{2}(\tau )+\sigma_{QN}^{2}(\tau )+\sigma_{RR}^{2}(\tau )+\cdots$$

The Allan variance of five random errors, such as quantization noise, angular random walk, zero bias instability, angular rate random walk, and rate slope, and the slope in the double logarithmic curve are shown in Table 1. The least squares method is used to fit the double logarithmic curve of Allan standard deviation and correlation time, and the error coefficients of five kinds of random errors are shown in Table 1. Because the rate slope is the random error of the long correlation time, this paper does not analyze it, so the error coefficients of the rate slope of the three gyroscopes are not listed in Table 1.

## 3. Conventional Virtual Gyroscope Technology

**Figure 4:**
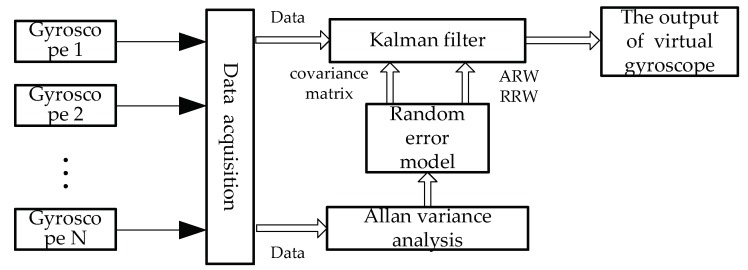
The process of the virtual gyroscope technology.

The conventional virtual gyroscope technology mainly uses the correlation between the MEMS gyroscope random error to carry on the data fusion, which can improve the output precision of the virtual gyroscope. The specific process of the virtual gyroscope technology is shown in Figure 4. First, the static random drift data of each MEMS gyroscope is collected. The random error characteristics of each MEMS gyroscope are obtained by analyzing the static random drift data with the Allan variance analysis method. After that, the random error model of MEMS gyroscope array with angular random walk and angular rate random walk is set up, and the corresponding Kalman filter is designed to fuse the data to compensate the random drift of the MEMS gyroscope.

### 3.1. Random Error Model of MEMS Gyroscope

The error of MEMS gyroscope is mainly divided into the deterministic error and the random error. The deterministic error can be compensated by a reasonable calibration test, and the compensation of random error is more difficult. The random error of MEMS gyroscope array is usually modeled as Equation (4) in conventional virtual gyroscope technology [4].

(4) $$\left\{ \begin{array}{l} y=\omega +b+n\\ \dot{b}=\varpi \end{array}\right.$$

In the equation, the *y* indicates the rate of the gyroscope output angle; ω represents the real angular rate of MEMS gyroscope detection; *b* represents angular rate random walk and it can be represented as random walk ϖ d riven noise; *n* is angular random walk. The error model of the MEMS gyroscope array consisting of three MEMS gyroscopes is shown in Equation (5).

(5) $$\dot{B}={\left[\begin{array}{ccc} \varpi_{1} & \varpi_{2} & \varpi_{3} \end{array}\right]}^{T}$$

(6) Y=I⋅ω+B+V

In the equation, $Y={\left[\begin{array}{ccc} y_{1} & y_{2} & y_{3} \end{array}\right]}^{T}$, $B={\left[\begin{array}{ccc} b_{1} & b_{2} & b_{3} \end{array}\right]}^{T}$, $V={\left[\begin{array}{ccc} n_{1} & n_{2} & n_{3} \end{array}\right]}^{T}$, $I={\left[\begin{array}{ccc} 1 & 1 & 1 \end{array}\right]}^{T}$.

### 3.2. Design of Kalman Filter

According to the random error model of MEMS gyroscope, the Kalman filter is designed to carry out data fusion of three MEMS gyroscopes. The angular rate random walk and real angular rate are selected as state vector of the Kalman filter, that is $X={\left[\begin{array}{cc} \begin{array}{ccc} b_{1} & b_{2} & b_{3} \end{array} & \omega \end{array}\right]}^{T}$. The real angular rate will not be absolutely equal to 0 due to external environment and other factors in static state. Therefore, the real angular rate ω is modeled as white noise driven by $\varpi_{\omega }$.

According to Equation (5), the Kalman filter state equation of MEMS gyroscope array is as shown in Equation (7). According to Equation (6), the measurement equation of the system is shown in Equation (8).

(7) $$\dot{X}=\Phi X+\Gamma W$$

(8) Y=HX+V

In the equation, Φ=0, Γ=I, $W={\left[\begin{array}{cccc} \varpi_{1} & \varpi_{2} & \varpi_{3} & \varpi_{\omega } \end{array}\right]}^{T}$, $H=\left[I_{3\times 3}\vdots I_{3\times 1}\right]$. $I_{3\times 3}$ represents the unit matrix of 3 rows and 3 columns, $I_{3\times 1}$ represents the unit column vector of 3 rows and 1 columns. The Equations (9) and (10) can be obtained by the discretization of the Equations (7) and (8).

(9) $$X_{k}=\Phi_{k/k-1}X_{k-1}+\Gamma_{k-1}W_{k-1}$$

(10) $$Y_{k}=H_{k}X_{k}+V_{k}$$

In the equation, $\Phi_{k/k-1}=I$, $\Gamma_{k-1}=T\cdot I$, *T* represents the period of discretization, $W_{k-1}$ is the drive white noise sequence, $V_{k}$ is the measurement noise sequence. The driving noise and the measurement noise meet the following requirements.

(11) $$\left\{ \begin{array}{l} E[W_{k}]=0,\mathrm{Cov}[W_{k},W_{j}]=Q_{k}\delta_{kj}\\ E[V_{k}]=0,\mathrm{Cov}[V_{k},V_{j}]=R_{k}\delta_{kj}\\ \mathrm{Cov}[W_{k},V_{j}]=E[W_{k}V_{j}^{T}]=0 \end{array}\right.$$

If there is correlation between the gyroscopes in the MEMS gyroscope array, there is correlation between the gyroscope random errors. The correlation coefficient ρ is used to characterize the correlation degree between random errors of different gyroscopes, −1≤ρ≤1, ρ≠0. A covariance of the driving noise is as follows.

(12) $$Q_{k}=\left[\begin{array}{cc} Q_{k}^{\prime } & 0_{3\times 1}\\ 0_{1\times 3} & {\sigma^{\prime }}^{2} \end{array}\right]$$

$$Q_{k}^{\prime }=\left[\begin{array}{ccc} \sigma_{1}^{2} & \rho_{12}\sqrt{\sigma_{1}^{2}\sigma_{2}^{2}} & \rho_{13}\sqrt{\sigma_{1}^{2}\sigma_{3}^{2}}\\ \rho_{21}\sqrt{\sigma_{2}^{2}\sigma_{1}^{2}} & \sigma_{2}^{2} & \rho_{23}\sqrt{\sigma_{2}^{2}\sigma_{3}^{2}}\\ \rho_{31}\sqrt{\sigma_{3}^{2}\sigma_{1}^{2}} & \rho_{32}\sqrt{\sigma_{3}^{2}\sigma_{2}^{2}} & \sigma_{3}^{2} \end{array}\right]$$

In the equation, $\sigma_{i}^{2}$ (*i* = 1, 2, 3) represents the RRW noise variance of the *i* gyroscope, $\rho_{ij}$ (*i*, *j* = 1, 2, 3) indicates the correlation between the *i* gyroscope and the *j* gyroscope, ${\sigma^{\prime }}^{2}$ represents the variance of the noise $\varpi_{\omega }$. $\rho_{ij}$ can be obtained by solving the cross correlation coefficient between the static random drift data of different gyroscopes. $\sigma_{i}^{2}$, ${\sigma^{\prime }}^{2}$ can be obtained by Allan variance. Similarly, the measurement noise covariance matrix is solved. The output data of 3 MEMS gyroscopes are fused by using Kalman filter technology, and the output of virtual gyroscope is obtained, which is recorded as $\text{\{}x_{t}\text{\}}$.

**Figure 5:**
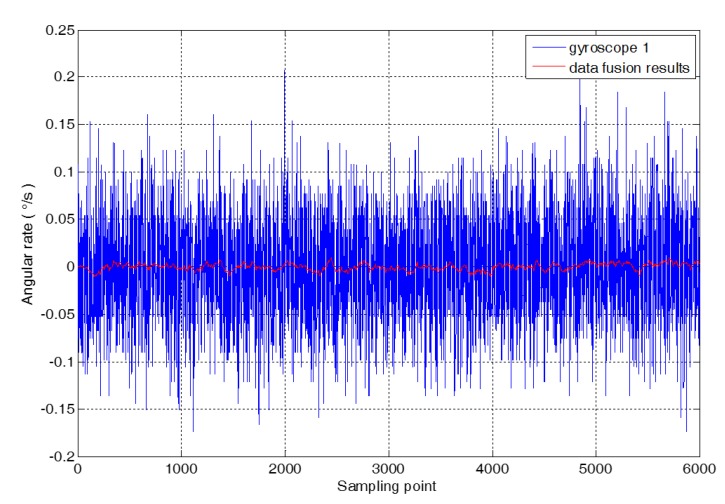
The results of random drift and data fusion.

The correlation coefficient between the gyroscopes is shown in Table 2. There are 6000 static random drift data of three gyroscopes selected as the research object, and the random error model of MEMS gyroscope array is combined with the Kalman filter technology to fuse the data of 3 gyroscopes. The comparison between the fusion result and the original random drift of gyroscope 1 is shown in Figure 5.

## 4. Virtual Gyroscope Technology Based on ARMA Model

The conventional virtual gyroscope technology only considers angular random walk and angular rate random walk when modeling MEMS gyroscope array random error. Because the random error model ignores many other random noise terms, the accuracy of the model is relatively low. In this paper, the ARMA model is used to model the output of the conventional virtual gyroscope, and the random error model of the MEMS gyroscope array is improved, and the corresponding Kalman filter is designed to achieve the data fusion of the MEMS gyroscope. This method can further improve the static compensation ability of the virtual gyroscope technology.

### 4.1. Modeling of ARMA Model

The ARMA model uses the form of random difference equation, which can quantitatively describe the linear relationship between the observed data, and the ARMA model has the function of predicting the time series. The ARMA model is used to model the time series of the virtual gyroscope output, and the accurate prediction of virtual gyroscope output is realized. For time series $\text{\{}x_{t}\text{\}}$, the ARMA model is shown in Equation (13).

(13) $$x_{t}+\sum_{i=1}^{p}m_{i}x_{t-i}=\sum_{j=1}^{q}r_{j}\varepsilon_{t-j}+\varepsilon_{t}$$

In the equation, $\varepsilon_{t}$ is a white noise with a variance of σ. σ is the residual variance of the fitting results of the high order AR model. $m_{i}$, $r_{j}$ is the model parameters. For a time series with a sample length of *N*, the ARMA model can be expressed as Equation (14).

(14) $$X^{\prime }=H^{\prime }\theta +\varepsilon$$

(15) $$X^{\prime }={\left[\begin{array}{cccc} x_{p+1} & x_{p+2} & \cdots & x_{N} \end{array}\right]}^{T}$$

(16) $$H^{\prime }=\left[\begin{array}{cccccc} -x_{p} & \cdots & -x_{1} & \varepsilon_{p} & \cdots & \varepsilon_{p+1-q}\\ -x_{p+1} & \cdots & -x_{2} & \varepsilon_{p+1} & \cdots & \varepsilon_{p+2-q}\\ \vdots & \vdots & \vdots & \vdots & \vdots & \vdots \\ -x_{N-1} & \cdots & -x_{N-p} & \varepsilon_{N-1} & \cdots & \varepsilon_{N-q} \end{array}\right]$$

(17) $$\theta ={\left[m_{1}\cdots m_{p}\;r_{1}\;\cdots r_{q}\right]}^{T}$$

The multiple regression theory is used to solve the Equation (14) and the least squares estimation of θ is:

(18) $$\theta ={\left({H^{\prime }}^{T}H^{\prime }\right)}^{-1}{H^{\prime }}^{T}X^{\prime }$$

The output of virtual gyroscope is $\text{\{}x_{t}\text{\}}$, which can be described as the Equation (19) with the determination of model parameters by Equation (18).

(19) $$x_{t}=m_{1}x_{t-1}+\cdots +m_{p}x_{t-p}+r_{1}\varepsilon_{t-1}+\cdots +r_{q}\varepsilon_{t-q}+\varepsilon_{t}$$

**Figure 6:**
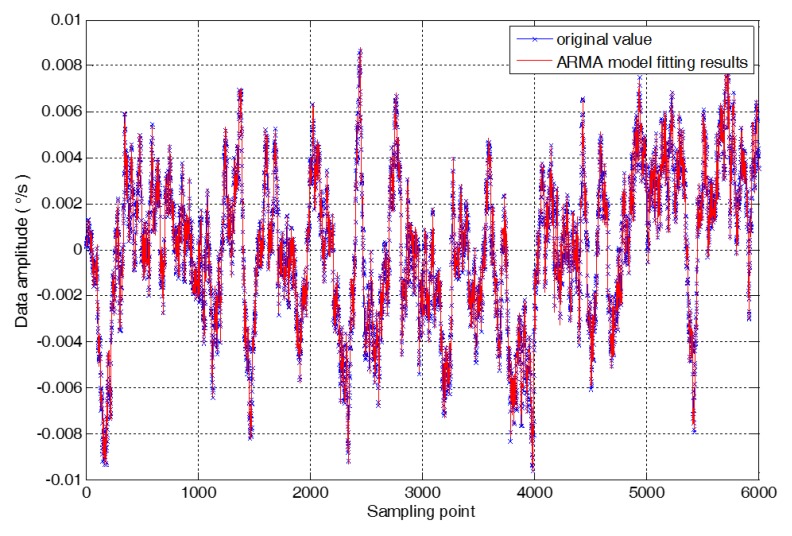
The original signal and the modeling result of the ARMA model.

The high order ARMA model can better describe the output characteristics of the virtual gyroscope. However, the high order ARMA model will greatly increase the dimension and computation of the filter. The ARMA model has a 2-order autoregressive model and 1 order moving average model, which is ARMA (2,1) model. The ARMA (2,1) model is chosen to model the time series $\text{\{}x_{t}\text{\}}$, which is the output of virtual gyroscope. The comparison between the original signal of virtual gyroscope output and the modeling results is shown in Figure 6. In the Equation (19), the model parameters are as follows. $m_{1}$ = 0.66177, $m_{2}$ = 0.32011, and $r_{1}$ = 0.62258, $\sigma^{2}=$ 2.60829 × 10^−8^.

### 4.2. Improved Random Error Model

The random error model in reference [2,3,4,5,6,7,8,9,10,11,12,13,14,15] only considers two random drift errors of angular random walk and angular rate random walk, neglecting the multiple random errors, and the virtual gyroscope technology can’t achieve the compensation of all random error. Therefore, the improved random error model of the MEMS gyroscope is established by combining Equations (4) and (19). The improved random error model is as shown in Equation (20).

(20) $$\left\{ \begin{array}{l} y=\omega +b+x_{t}+n\\ \dot{b}=\varpi \\ x_{t}=m_{1}x_{t-1}+m_{2}x_{t-2}+r_{1}\varepsilon_{t-1}+\varepsilon_{t} \end{array}\right.$$

In the equation, *y* indicates the rate of the gyro output angle; ω represents the real angular rate of MEMS gyro detection; *b* represents angular rate random walk and it can be represented as random walk ϖ driven noise; *n* is angular random walk and $x_{t}$ is a compensation residual that is the output of the virtual gyroscope. $\varepsilon_{t}\sim \mathrm{NID}(0,\sigma )$, σ is the variance of modeling residuals.

### 4.3. Improved Kalman Filter Design

According to the modified random error model, the Kalman filter is designed for data fusion of MEMS gyroscope array. The state vectors of the Kalman filter are as follows:

(21) $$X={\left[\begin{array}{ccc} \begin{array}{cccc} b_{1} & b_{2} & b_{3} & x_{t} \end{array} & x_{t-1} & \omega \end{array}\right]}^{T}$$

The observation is the actual output of three MEMS gyroscope. The discrete state equation is as Equation (22), which is obtained by combining Equations (9) and (20). Measurement equation is as shown in Equation (23).

(22) $$X_{k}=F_{k/k-1}X_{k-1}+\Gamma_{k-1}W_{k-1}$$

(23) $$Z_{k}=H_{k}X_{k}+V_{k}$$

In the equation,

$$F_{k/k-1}=\left[\begin{array}{cc} I_{3\times 3} & 0_{3\times 3}\\ 0_{3\times 3} & \begin{array}{ccc} m_{1} & 1 & 0\\ m_{2} & 0 & 0\\ 0 & 0 & 1 \end{array} \end{array}\right],\;\Gamma_{k-1}=\left[\begin{array}{cc} T\cdot I_{3\times 3} & 0_{3\times 3}\\ 0_{3\times 3} & \begin{array}{ccc} 1 & 0 & 0\\ r_{1} & 0 & 0\\ 0 & 0 & T \end{array} \end{array}\right],\;H_{k}=\left[\begin{array}{cc} I_{3\times 3} & \begin{array}{ccc} 1 & 0 & 1\\ 1 & 0 & 1\\ 1 & 0 & 1 \end{array} \end{array}\right].$$

$$Z_{k}={\left[\begin{array}{ccc} y_{1}(k) & y_{2}(k) & y_{3}(k) \end{array}\right]}^{T}$$

The driving noise $W_{k}$ and measurement noise $V_{k}$ satisfy the conditions as the Equation (24). $Q_{k}$ is a driving noise covariance matrix, and $Q_{k}$ is as the Equation (25). In the equation, $\sigma^{2}$ is the variance of noise $\varepsilon_{t}$, ${\sigma^{\prime }}^{2}$ is the variance of noise ϖ. $R_{k}$ is the measurement noise covariance matrix, which is the same as the $R_{k}$ in Section 3.2.

(24) $$\left\{ \begin{array}{l} E[W_{k}]=0,\mathrm{Cov}[W_{k},W_{j}]=Q_{k}\delta_{kj}\\ E[V_{k}]=0,\mathrm{Cov}[V_{k},V_{j}]=R_{k}\delta_{kj}\\ \mathrm{Cov}[W_{k},V_{j}]=E[W_{k}V_{j}^{T}]=0 \end{array}\right.$$

(25) $$Q_{k}=\left[\begin{array}{cc} Q_{k}^{\prime } & 0_{3\times 3}\\ 0_{3\times 3} & \begin{array}{ccc} \sigma^{2} & & \\ & \sigma^{2} & \\ & & {\sigma^{\prime }}^{2} \end{array} \end{array}\right]$$

The data fusion of three MEMS gyroscopes is carried out by using the designed Kalman filter to further improve the compensation ability of the virtual gyroscope.

## 5. Test Analysis

**Figure 7:**
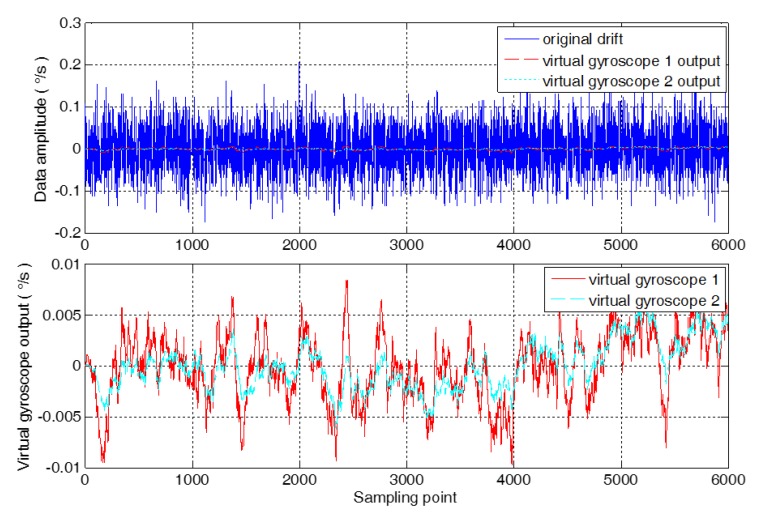
The comparison of the output of the virtual gyroscope.

Data fusion of MEMS gyroscope array is carried out by using a Kalman filter based on an improved random error model. When the sample length of the fusion data is 6000, the comparison results between the output of virtual gyroscope (simply marked “virtual gyroscope 1”) based on the conventional random error model and the output of virtual gyroscope (simply marked “virtual gyroscope 2”) based on the improved random error model is shown in Figure 7.

It is shown in Figure 7 that the method proposed in this paper can further compensate the compensation residual of the virtual gyroscope, so the compensation effect of the virtual gyroscope 2 is obviously better than the compensation effect of the virtual gyroscope 1. Use 1σ standard deviation as an evaluation criterion to determine the compensation performance of different compensation algorithms. The 1σ standard deviation of compensation results of virtual gyroscope 1 is 3.3657 × 10^−3^°/s. And the 1σ standard deviation of compensation results of virtual gyroscope 2 is 2.3518 × 10^−3^°/s, which reduced by 1.4311 times compared with virtual gyroscope 1.

**Figure 8:**
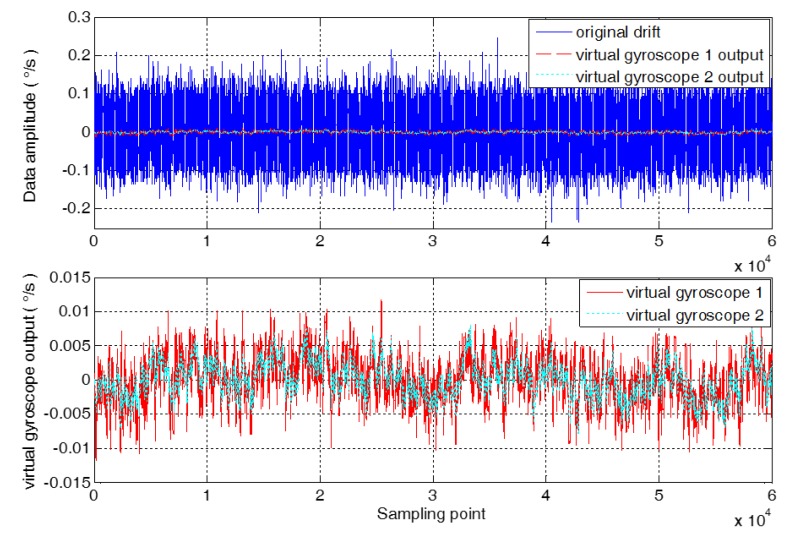
The comparison of the output of the virtual gyroscope.

In order to further verify the feasibility of the proposed method, the long-time sampling of the MEMS gyroscope array is carried out, and 60,000 sampling data are obtained. The data fusion of the MEMS gyroscope array is carried out by using virtual gyroscope 1 and virtual gyroscope 2, respectively. The output contrast results of the virtual gyroscope are obtained as shown in Figure 8.

As seen in Figure 8, the algorithm is modeled for the conventional virtual gyroscope compensation residual error, and the random error model and Kalman filter are improved to increase the compensation capability of the virtual gyroscope. The 1σ standard deviation between the compensation result of virtual gyroscope 1 and virtual gyroscope 2 is calculated, respectively. The 1σ standard deviation of compensation results of virtual gyroscope 1 is 3.3124 × 10^−3^°/s. And the 1σ standard deviation of compensation results of virtual gyroscope 2 is 2.3277 × 10^−3^°/s, which reduced by 1.4230 times compared with virtual gyroscope 1.

**Figure 9:**
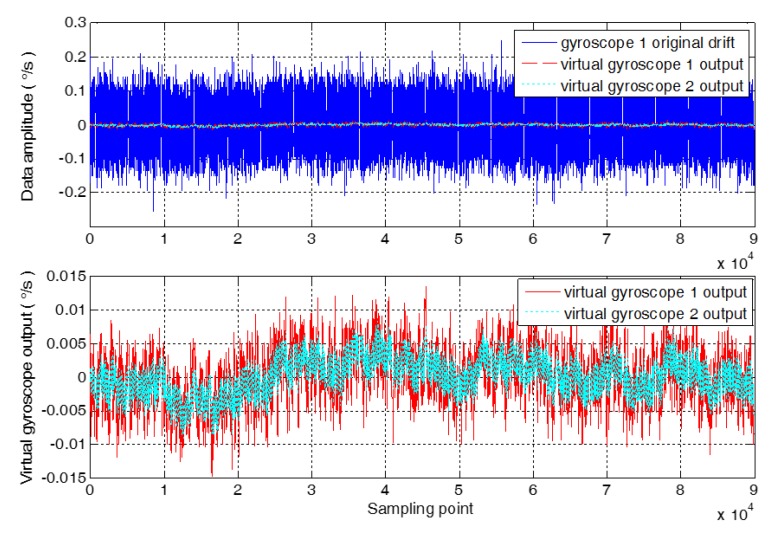
The comparison of the output of the virtual gyroscope.

There are 90,000 sets of static data collected to carry out the third verification tests using the proposed method. The virtual gyroscope 1 method and the virtual gyroscope 2 method are used for data fusion, respectively, and the fusion results and their comparison results are shown in Figure 9. The 1σ standard deviation of the two data fusion results is obtained to determine the accuracy of the fusion results. The 1σ standard deviation of compensation results of virtual gyroscope 1 is 3.7535 × 10^−3^°/s. And the 1σ standard deviation of compensation results of virtual gyroscope 2 is 2.4656 × 10^−3^°/s, which reduced by 1.5223 times compared with virtual gyroscope 1. In summary, the feasibility of the proposed method is verified.

## 6. Conclusions

In this paper, the conventional virtual gyroscope technology is used to model the random error of three MEMS gyroscopes, and the data fusion is carried out through Kalman filter, and the output of virtual gyroscope is obtained. Then the ARMA model is used to model the output of virtual gyroscope, and combine the ARMA model and conventional random error model to improve the random error model. The Kalman filter based on improved random error model is designed for data fusion of three MEMS gyroscopes. The experimental results show that the virtual gyroscope technology based on ARMA model can further improve the compensation ability of conventional virtual gyroscope technology and the 1σ standard deviation of the output has been reduced by 1.4 times compared with conventional virtual gyroscope technology.

## Figures and Tables

**Table 1:** The Allan variance of five main random errors in MEMS gyroscopes.

Error Term	Allan Variance	Slope	Coefficient	Unit	Gyro 1	Gyro 1	Gyro 1
Quantization noise	$\sigma_{QN}^{2}(\tau )=\frac{3Q^{2}}{\tau^{2}}$	−1	Q	deg	0.00821	0.00726	0.00707
Angular random walk	$\sigma_{ARW}^{2}(\tau )=\frac{N^{2}}{\tau }$	−1/2	N	deg/h^1/2^	0.355	0.315	0.314
Zero bias instability	$\sigma_{BI}^{2}(\tau )=\frac{B^{2}\cdot 2ln2}{\pi }$	0	B	deg/h	10.318	7.790	8.533
Angular rate random walk	$\sigma_{RRW}^{2}(\tau )=\frac{P^{2}\tau }{3}$	1/2	P	deg/h^3/2^	52.323	120.310	61.357
Rate Ramp	$\sigma_{RR}^{2}(\tau )=\frac{R^{2}\tau^{2}}{2}$	1	R	deg/h^2^	-	-	-

**Table 2:** The correlation coefficients between each gyroscopes.

Gyroscope Number	Gyro 1	Gyro 2	Gyro 3
Gyro 1	1	−1.0582 × 10^−4^	1.6087 × 10^−3^
Gyro 2	−1.0582 × 10^−4^	1	1.3325 × 10^−2^
Gyro 3	1.6087 × 10^−3^	1.3325 × 10^−2^	1
